# The Crisis of Macrolide Resistance in Pneumococci in Latin America

**DOI:** 10.4269/ajtmh.23-0913

**Published:** 2024-07-30

**Authors:** Carolina Viteri-Dávila, Diana Morales-Jadán, Aidan Creel, Ana G. Jop Vidal, Xavier M. Boldo, Ismar A. Rivera-Olivero, Consuelo Bautista-Muñoz, Babek Alibayov, Miguel Ángel Garcia-Bereguiain, Jorge E. Vidal

**Affiliations:** ^1^ One Health Research Group, Universidad de Las Américas, Quito, Ecuador;; ^2^ Summer Undergraduate Research Experience Program, School of Graduate Studies in the Health Sciences, University of Mississippi Medical Center, Jackson, Mississippi;; ^3^ Department of Cell and Molecular Biology, University of Mississippi Medical Center, Jackson, Mississippi;; ^4^ Research Center, Health Sciences Academic Division, Juarez Autonomous University of Tabasco, Villahermosa, Mexico;; ^5^ Laboratory of Biotechnology, Colegio de Postgraduados Campus Tabasco, Cardenas, Mexico;; ^6^ Center for Immunology and Microbial Research, University of Mississippi Medical Center, Jackson, Mississippi

## Abstract

Macrolide antibiotics are recommended for the treatment of pneumococcal pneumonia and invasive pneumococcal disease (IPD). Prior to 2000, ∼10% of *Streptococcus pneumoniae* strains isolated from IPD cases in Latin American countries were resistant to macrolides. The mechanism of resistance to macrolides was associated mainly with the efflux pump known as the macrolide efflux genetic assembly, since most pneumococcal strains carried the *mef(A/E)* gene, whereas <6% strains carried both the methylase gene *ermB* and *mef(A/E)*. In the first decade of this century, a significant increase in the prevalence of macrolide resistance was observed in pneumococcal strains in both Mexico and Peru. Approximately 30% of *S. pneumoniae* strains in these countries were already resistant to erythromycin, while the prevalence in Colombia, Argentina, and Brazil remained below 10%. During the last decade, we have been experiencing a worrisome increase in pneumococcal strains carrying resistance to macrolides, with a prevalence of up to 80% for resistance to erythromycin. The mechanism for disseminating macrolide resistance has evolved. Currently, more than 55% of invasive *S. pneumoniae* macrolide-resistant strains carry both the *ermB* and the *mef(A/E)*/*mel* genes. Lessons learned from the current macrolide resistance crisis in Latin America can inform interventions in other regions.

## INTRODUCTION

Antibiotics have saved millions of lives and have contributed to human life span growth since they were discovered and used to treat infectious diseases in the first half of the 20th century. However, the increasing rate of antibiotic resistance throughout the world has created a serious public health threat. The rising prevalence of antibiotic resistance has been associated with nonadherence, improper antibiotic therapy, and misdiagnosis of the etiology, followed by unnecessary prescription of antibiotics for diseases caused by nonsusceptible organisms.^1^^–^^3^ Another important activity contributing to the increased resistance in Latin America is the massive veterinary use in the farming industry, although we lack metrics on this.

In most Latin American countries, antibiotic treatment options are often empirical due to the limited availability of antibiotic susceptibility tests or to delayed delivery of test results at government-sponsored health care centers. Self-prescription of macrolides and their availability over the counter have probably contributed to the increase of antibiotic resistance. There is progress in this regard made by Brazil and Mexico, two countries that already banned over-the-counter sales of antibiotics in 2011 and 2012, respectively. Finally, the lack of adherence to antibiotic treatment caused by self-prescription led by the local marketing of macrolides, including detailed instructions for their administration, has greatly contributed to this widespread practice.^4^^–^^8^

*Streptococcus pneumoniae* (or pneumococcus) is a Gram-positive bacterium that forms part of the microbiota of the human nasopharynx. Pneumococci are also opportunistic pathogens and the world’s leading cause of death in children under 5 years of age due to community-acquired pneumonia (CAP).^9^ Acute bacterial respiratory infections kill more than one million children less than 5 years old in developing countries each year, and it is estimated that ∼40% of these deadly infections are caused by pneumococci.^10^^,^^11^

The current antibiotic therapy for respiratory disease caused by Gram-positive bacteria, such as pneumococcal disease, includes the macrolide antibiotics erythromycin, azithromycin, and clarithromycin. These three macrolides are used worldwide. According to the guidelines for the antibiotic treatment of CAP, issued by the Infectious Diseases Society of America and the American Thoracic Society, macrolides are recommended as the first-line empirical therapy in outpatients diagnosed with CAP with no associated comorbidities, as long as the prevalence of *S. pneumoniae* strains bearing resistance to macrolides in the area is <25%.^6^^,^^12^^,^^13^

A study conducted in the United States in 2011 demonstrated that macrolides were one of the most prescribed antibiotics in that year, with 190 prescriptions per 1,000 cases of infections. The increased prescription of macrolides may have caused an increase in isolation of *S. pneumoniae* strains with resistance to these antibiotics. For example, studies conducted during a period of high macrolide prescription rates in the last decade revealed that between 20% and 40% of pneumococcal strains isolated from invasive pneumococcal disease (IPD) cases in the United States exhibited resistance to macrolides.^14^^,^^15^ A more recent study by Paukner et al.^16^ highlights that in different geographic regions of the United States (i.e., West North Central, Middle Atlantic, or East South Central), the prevalence of *S. pneumoniae* with macrolide resistance has already surpassed the recommended prevalence burden of <25% for prescribing a macrolide. Surveillance studies are important to monitor changing trends in macrolide resistance for an effective CAP antibiotic therapy.^2^^,^^15^^,^^17^^,^^18^ In this article, we provide a comprehensive literature review of macrolide resistance in pneumococcal disease with a special focus on the epidemiology of pneumococcal strains bearing macrolide resistance in Latin American countries.

## OVERVIEW OF THE GLOBAL SPREAD OF MACROLIDE RESISTANCE IN *S. PNEUMONIAE*

To the best of our knowledge, the first description of resistance to erythromycin generated in vitro in *S. pneumoniae *strains was published in 1952.^19^ Identification of in vitro-generated resistance was followed by the isolation of clinical strains bearing resistance to this macrolide in patients with bronchitis and the isolation of a resistant strain causing empyema in a patient with lung cancer. Along the same lines, Canada first reported macrolide-resistant pneumococci in 1967. In the 1980s, surveillance studies showed a trend of increasing macrolide resistance worldwide. For example, in Belgium, the isolation of pneumococcal strains with resistance to macrolides increased from a prevalence of ∼1.1% in 1983 to ∼13% in 1988.^19^

A study conducted by Cilloniz et al.^20^ from 2000 to 2013 in Spain reported macrolide resistance in 22% of *S. pneumoniae *strains. Of note, patients infected with macrolide-resistant strains were less likely to have bacteremia, pulmonary complications, and shock.^20^ In Greece and Turkey, two neighboring countries, the prevalence rates have been estimated at ∼29% and ∼2%, respectively. According to different studies, the prevalence of resistance also varies greatly in Western Europe, ranging from 48% to 58% in France to ∼17% in Germany and ∼9% in England.^21^

In Asia, the rate of macrolide resistance in *S. pneumoniae *strains is worrisome. A recent study showed that ∼70% of clinical isolates of *S. pneumoniae *were resistant to macrolides in Asian countries, and the proportion of isolates of *S. pneumoniae *that were not susceptible to azithromycin was ∼82.0%.^22^ The highest prevalence of macrolide resistance has been reported in the Far East region of Asia, including Taiwan (98%), South Korea (88%), Japan (78%),^22^ and China (98%).^23^

## MECHANISM OF ACTION OF MACROLIDE ANTIBIOTICS

The term macrolide describes natural and semisynthetic drugs with a macrocyclic lactone ring of 12 or more elements. Macrolides block the synthesis of proteins by reversible binding of the 50S bacterial ribosome with a specific target in the 23S ribosomal RNA (rRNA) molecule and various ribosomal proteins such as L4 and L22.^16^^,^^24^^,^^25^ All macrolide antibiotics, from the prototype of this class, erythromycin, to those of later generations as ketolides (including telithromycin) with wider spectra of activity against pathogens, were developed from 14-membered macrolides. These drugs contain a keto group instead of the cladinose residue at position 3 of the lactone ring and have alkyl–aryl side-chains. The 14-, 15-, and 16-membered macrolides are a widely used family of antibiotics. They have excellent tissue penetration and antimicrobial activity, mainly against Gram-positive cocci and atypical pathogens. The Food and Drug Administration approved the use of macrolide antibiotics for a wide variety of bacterial infections, such as sexually transmitted infections, chlamydial infections, classical and atypical pneumonia, sinusitis, pharyngitis, and tonsillitis. Macrolides are also used to treat uncomplicated skin infections and otitis media.^24^^,^^26^^–^^30^

**Figure 1.**
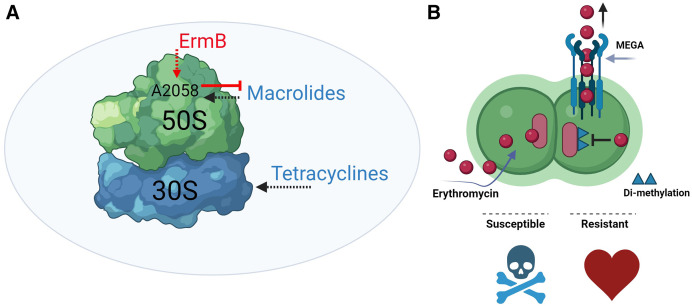
Target of macrolides and most common resistance mechanism to macrolides. (**A**) Macrolide antibiotics target the 50S subunit of the 70S bacterial ribosome, whereas tetracyclines target the 30S subunit. ErmB dimethylates an adenine residue (A2058), preventing binding of macrolides. (**B**, left) Macrolides bind to their target sites in the ribosomes of susceptible pneumococci. (**B**, right) Macrolide resistance, facilitated by the macrolide efflux genetic assembly (Mega) mechanism, involves an efflux pump located on the bacterial surface that pumps out the macrolide, making pneumococci resistant. Additionally, a methylase encoded typically by the *ermB* gene methylates binding sites in the 50S subunit, inhibiting the binding of macrolides like erythromycin to their targets. This process renders pneumococci resistant.

The mechanism of action of macrolides has been studied for more than 60 years; all macrolides inhibit bacterial protein synthesis (Figure 1A). The precise mechanism leading to protein synthesis inhibition depends on the chemical structure of the drug but includes the following: 1) inhibition of the progression of the incipient peptide chain in the course of initial translation,^31^ 2) inhibition of peptide-bond synthesis,^31^ 3) stimulation of dissociation of peptidyl-transfer RNA (tRNA) from ribosomes during translocation,^32^ and 4) inhibition of 50S ribosomal subunit assembly.^33^

Recent studies using high-resolution crystal structures, X-ray crystallography, cryo-electron microscopy, biochemical studies, and genome-wide analysis revealed that macrolides act also as regulators of translation by occupying a site within the nascent peptide exit tunnel adjacent to the peptidyl transferase center.^34^^–^^38^

## MECHANISM OF RESISTANCE TO MACROLIDES

*Streptococcus pneumoniae *macrolide resistance is attributed mainly to modification of the macrolide target in the bacterial ribosome and to active efflux mediated by the macrolide efflux genetic assembly (Mega), an efflux pump encoded by a ∼5.5- or 5.4-kb element, *mef(A/E)*, and *mel* (Figure 1B).^39^ Modification of the bacterial ribosome target occurs through at least two different mechanisms, point mutations and methylation in the target site. A mutation(s) in 23S rRNA or L4 or L22 ribosomal protein genes *rplD* and *rplV*, respectively, has been associated with nonsusceptibility to macrolides, whereas another common mechanism involves the methylation of the 23S rRNA by methyltransferases encoded by *ermB* and rarely *ermTR*.^40^

The *ermB* gene encodes an adenine-specific N-methyltransferase that is responsible for the ribosomal dimethylation in the N6 position of the A2058 residue of the 23S rRNA (*Escherichia coli* numbering [Figure 1A]); this methylation prevents antibiotic binding.^41^^,^^42^ In *S. pneumoniae *strains with phenotypic resistance to macrolides, ribosomal modification is also caused by other methylases encoded by the *ermA* and *ermTR* genes; the genes encoding these methylases are less frequently detected than *ermB*.^40^^,^^42^ Resistance conferred by the dimethylation of A2058 comes with a fitness cost, including disturbance of the translation of some proteins. Dimethylation of the 23S rRNA by Erm methyltransferases confers resistance to other structurally unrelated antibiotics such as lincosamides and streptogramin B, known as the MLS_B_ phenotype.^42^^,^^43^

Another mechanism conferring macrolide resistance to *S. pneumoniae *strains is a two-component Mega element encoded by the *mef*(*A*, *E*, or *I*)/*mel* (listed also as *msrD*) operon.^44^ *mef(A)* is most common in strains isolated in Europe and the United States,^45^ whereas *mef(E)* is commonly carried by strains isolated in Africa, Asia, and the United States and *mefI* has been detected only in *S. pneumoniae *strains isolated in Italy.^46^

Transcription of *mef(A/E)/mel* genes is driven by a single promoter that is induced by macrolide and resulting in expression of the efflux pump that in turn results in an efflux of the antibiotic. *Streptococcus pneumoniae* bacteria producing Mega have been shown to have an M phenotype, which is resistant to 14- and 15-membered macrolides but susceptible to lincosamides and streptogramin B.^42^ Usually, *S. pneumoniae *strains are more resistant due to a dual macrolide resistance genotype, i.e., strains carrying both *ermB* and *mef(E)/mel* genes.^42^^,^^44^

## THE RISE OF MACROLIDE RESISTANCE IN LATIN AMERICA BEFORE THE YEAR 2000

Studies published before the year 2000 presented only the overall prevalence of macrolide resistance in *S. pneumoniae *strains. However, those published around the beginning of the year 2000 and after provided not only the overall prevalence of macrolide resistance but also the prevalence of specific mechanisms of macrolide resistance carried by the strains. Additionally, the pneumococcal conjugate vaccine (PCV) was licensed in the United States in 2000, and a number of Latin American countries introduced the PCV into their national vaccination programs in the same decade. Therefore, we have divided the description of macrolide resistance in *S. pneumoniae *isolated in Latin American countries into two periods, before and after the year 2000.

One of the first reports found in the literature with pneumococcal isolates bearing resistance to macrolides is from Brazil. Authors analyzed antibiotic resistance of pneumococcal strains isolated from 1988 through 1992 and found that only 6.1% of isolates expressed intermediate resistance to erythromycin.^47^ A subsequent study conducted in Brazil in 1996, which investigated the antibiotic resistance profile of pneumococcal strains isolated from blood cultures, revealed that most *S. pneumoniae *strains were susceptible to erythromycin (*N* = 50) whereas only one strain exhibited intermediate susceptibility.^48^

By the year 1998, a multicountry study, which included Argentina, Brazil, Chile, Colombia, Uruguay, and Mexico, was conducted by the Pan American Health Organization (PAHO).^49^ Investigators found a low prevalence of 6.4% macrolide resistance in *S. pneumoniae *isolated from invasive pneumococcal disease.^49^ A follow-up study by PAHO that included 4,105 *S. pneumoniae *isolates collected from 1993 through 1999 reported that 6.9% of isolates showed reduced susceptibility to erythromycin.^50^ Another study conducted in a similar setting, and time period, demonstrated low resistance to macrolides in *S. pneumoniae *strains isolated in the 1990s.^51^ Country-specific studies conducted prior to 2000 in Argentina, Brazil, Colombia, and Chile indicated a low prevalence of *S. pneumoniae *strains resistant to macrolides. These studies reported that ∼10% of *S. pneumoniae *strains isolated from IPD cases exhibited resistance to clarithromycin or erythromycin.^52^^–^^57^ Overall, the published evidence suggests that prior to the year 2000, most *S. pneumoniae *strains isolated from IPD in Latin American countries were susceptible to macrolides. A summary of the prevalence of macrolide resistance in *S. pneumoniae *strains before and after 2000 is shown in Table 1.

**Table 1:** Prevalence of macrolide resistance in *Streptococcus pneumoniae* strains in Latin America

Year(s)	Countries	*N*	Resistance (%)	Reference
1988–1992	Brazil	288	6.1	47
1996	Brazil	50	1	48
1993–1996	Argentina, Brazil, Chile, Colombia, Mexico, and Uruguay	326	6.4	49
1993–1999	Argentina, Brazil, Chile, Colombia, Mexico, and Uruguay	4,109	6.9	50
1999–2000	Argentina, Brazil, Mexico	518	15.3	58
2003–2004	Argentina	116	12.1	84
2003–2004	Brazil	308	6.2	84
2003–2004	Colombia	58	0	84
2003–2004	Mexico	105	27.6	84
2003–2004	Venezuela	33	33.3	84
2003–2004	Peru	54	27.8	84
2005	Mexico	122	>30	59
2006–2008	Argentina	563	20.4	96
2006–2008	Peru	313	33.5	71
2006–2008	Peru	101	24.8	71
2007–2012	Brazil	228	7.9	67
2008–2011	Colombia	134	4.8	97
2009–2010	Argentina	126	20.6	87
2010–2012	Brazil	159	8.2	68
2009–2011	Argentina	923	29.4	96
2009–2011	Peru	56	37.5	71
2012–2013	Argentina	489	24.1	96
2012–2013	Colombia	44	5.6	97
2014–2016	Argentina	454	23.8	96
2014–2019	Colombia	192	35.2	97
2016–2019	Peru	85	78.8	71
2017–2019	Argentina	369	35.2	96
2018–2019	Peru	208	50	71

## DISSEMINATION OF MACROLIDE RESISTANCE IN PNEUMOCOCCAL STRAINS OCCURRED IN THE DECADE OF 2000 IN LATIN AMERICAN COUNTRIES

In a study published in 2003, ∼15% of pneumococcal isolates collected from Brazil, Argentina, and Mexico at the beginning of the decade were resistant to erythromycin, with those strains isolated in Mexico yielding the highest prevalence of erythromycin resistance, at 31.2%.^58^ In this study, strains carrying *mef(E)* were more common and those carrying both *ermB* and *mef(E)* represented 1.3% of isolates. Another study conducted in Mexico City and published in 2005 isolated >30% of *S. pneumoniae *strains bearing resistance to erythromycin.^59^

Supporting evidence of a steady increase in macrolide resistance was observed in Peru. A 2-year multicenter study conducted from 2006 to 2008 reported erythromycin resistance in 24.8% of pneumococcal strains isolated from IPD cases. Another study conducted from 2009 to 2011 reported a further increase in resistance to 34.5% in *S. pneumoniae *strains isolated from adults and 37.5% in those from children.^60^^–^^62^ Additionally, a study screening healthy children found a prevalence of 26.3% or 28.9% of resistance to erythromycin or azithromycin, respectively, in strains isolated from the upper airways.^63^ In 2011, a similar prevalence of 22.4% of erythromycin resistance was observed in *S. pneumoniae *strains isolated from the nasopharynx of healthy children in the rural community of Cajamarca, Peru.^64^

Argentina also reported an increase in erythromycin resistance, from 9% in 1998 to 16% in 2002.^65^ The increase of resistance in Argentina was a particular one, given that a serotype 6B clone, related to the Poland(6B)-20 clone, was prevalent in Argentina in the decade of 2000 and that all isolates belonging to this clonal type were resistant to erythromycin.^52^ Unlike Mexico, Peru, and Argentina, other Latin American countries maintained a prevalence of macrolide resistance in single digits. For example, in Brazil, the prevalences of pneumococcal strains with resistance to erythromycin were 9.4% in a study spanning 1998 to 2004^66^ and ∼8% in studies conducted in 2007 to 2010, or 2010 to 2012, with strains carrying *mef(E)* being more prevalent.^67^^,^^68^ Another study in Brazil including 6,470 strains collected from 1993 to 2004 reported a prevalence of 6.2% for macrolide resistance.^69^ *Streptococcus pneumoniae *strains isolated in Colombia remained susceptible to macrolides through the year 2008, with a prevalence of erythromycin resistance of <6.9%.^57^^,^^70^

## CONSOLIDATED DISSEMINATION OF MACROLIDE RESISTANCE IN PNEUMOCOCCAL STRAINS FROM LATIN AMERICAN COUNTRIES

Over the past 12 years, a dramatic increase in macrolide resistance in *S. pneumoniae *has been observed not only in Latin American countries but also in other parts of the world. In Peru, the prevalence of macrolide resistance in carriage isolates was as high as 50%, while that of IPD isolates reached 78.8%.^71^ In this study conducted in Peru and where strains were stratified into three different periods, *S. pneumoniae *strains carrying the *ermB* gene accounted for >85% of macrolide-resistant isolates but strains carrying both *ermB* and *mef(E)* were more prevalent (>48% of prevalence in any given period) than those carrying only *ermB* or only *mef(E)*.^71^ Two different studies conducted in Brazil reported 42% (2014) or 28% (2017) resistance to erythromycin in invasive pneumococcal strains or strains isolated from healthy children, respectively.^72^^,^^73^ Overall, in 2010, >25% of resistance to macrolides was observed in Latin America.^74^^,^^75^

Specific data by country and year, where available, regarding resistance to azithromycin, erythromycin, and clarithromycin can be found in Table 2.

**Table 2:** Prevalence of resistance to erythromycin, azithromycin, or clarithromycin in *Streptococcus pneumoniae* strains

Countries	Antibiotic	Strains (*n*)	Resistance (%)	Year(s)	Reference
Argentina	Azithromycin	326	10	1997 – 2001	56
	Erythromycin	326	13.5		
	Clarithromycin	326	11.9		
Brazil	Azithromycin	497	8.6	1997 – 2001	56
	Erythromycin	497	11.5		
	Clarithromycin	497	10.8		
Chile	Azithromycin	533	13.3	1997 – 2001	56
	Erythromycin	533	12		
	Clarithromycin	533	12.8		
Peru	Azithromycin	572	28.9	2007–2009	63
	Erythromycin	572	26.3	2007–2009	
	Erythromycin	313*	33.5	2006–2008	71
	Erythromycin	101^†^	24.8	2006–2008	
	Erythromycin	56^†^	37.5	2009–2011	
	Azithromycin	208^‡^	50	2018–2019	
	Azithromycin	85^§^	78.8	2016–2019	
	Erythromycin	125	13.6	2009	64
	Erythromycin	125	22.4	2011	

* Carriage strains, pre-PCV13 introduction.

^†^ Invasive strains, pre-PCV13 introduction.

^‡^ Carriage strains, post-PCV13 introduction.

^§^ Invasive strains, post-PCV13 introduction.

## EFFECT OF THE PCV ON MACROLIDE RESISTANCE IN *S. PNEUMONIAE *STRAINS IN LATIN AMERICA

The introduction of the seven-valent PCV (PCV7) at the dawn of the 21st century has drastically altered the epidemiology of IPD. Globally, the PCV has also had several other salutary effects, such as diminishing the incidence of antibiotic-resistant *S. pneumoniae*. However, there are considerable regional disparities based on the type of vaccine used, schedules, and the circulation of specific serotypes, clones, and sequence types.^76^

Latin American countries began incorporating the PCV into their national vaccination schedules in 2007, with Costa Rica leading the way, followed by Mexico, Uruguay, and Peru in 2008, Brazil, Ecuador, El Salvador, Panama, and Nicaragua in 2010, Chile, Colombia, and Honduras in 2011, and Argentina, Guatemala, and Paraguay in 2012. Cuba initiated vaccination with its locally produced PCV. Venezuela introduced PCV7 in 2010 but only in private practice, while in July 2014, PCV13 was introduced, although its application has been intermittent over the years.^77^^–^^79^

Over the past several decades, there has been a notable increase in macrolide resistance among *S. pneumoniae *isolates in the region, despite the use of conjugate vaccines. In Argentina, the isolation of erythromycin-resistant *S. pneumoniae *strains significantly increased during the PCV13 period compared with that in the pre-PCV period. Moreover, during the PCV13 period, vaccine types (VTs) remained the primary contributors to all antibiotic and multidrug resistance, particularly serotypes 14 and 19A. This trend is largely due to the circulation of various clones and clonal replacement, likely associated with antibiotic-selective pressure resulting from the increased use of macrolides by over 200% from 2000 to 2015 in this country.^54^^,^^80^

This trend of increasing macrolide resistance has been observed not only in Argentina but also in other countries in the region, such as Peru, where macrolide resistance increased significantly after the introduction of PCV vaccination. The data reveal that resistance to macrolides rose from 24.8% in 2006–2008 to 78.8% in 2016–2019. The serotype associated with macrolide resistance is serotype 19A. Moreover, similar findings have been reported in Colombia and Costa Rica, indicating a regional pattern.^64^^,^^81^^–^^83^

The emergence of multidrug-resistant clones of serotype 19A is associated with the trends of antimicrobial resistance in Latin America. However, there is a diverse genetic background of the pneumococcal population in the Latin American countries, which requires continuous surveillance to understand the geographical differences and changes in spread of specific clones. It is crucial to comprehend the emergence of these resistant strains to effectively control their spread.^83^

## PREVALENCE OF MEGA AND METHYLASE-ENCODING GENES CARRIED BY STRAINS ISOLATED IN LATIN AMERICA

In a retrospective study conducted from 1994 through 2008 in Colombia, a low prevalence of macrolide-resistant *S. pneumoniae *strains (4.2%) was observed. Among these strains, 33% carried Mega [*mef(A)/mel* genes], while 60.39% carried the *erm(B)* gene, resulting in the MLB phenotype (Table 3). In a study conducted between 2005 and 2008, 30.7% of *S. pneumoniae *strains were positive for the *mef(E)* gene, and in 53.7% of the isolates, *erm(B)* was amplified. Peru reported a prevalence of 33.3% for strains positive for *mef(A/E)* and 53.3% for those carrying *erm(B)*; these strains were isolated in 2003 and 2004. In 2011, 11.9% of *S. pneumoniae *strains carried *mefA*, 10.31% were positive for *ermB*, and 11.9% carried *mefE* in Venezuela.^67^^,^^84^^–^^87^

**Table 3:** Prevalence of macrolide resistance genes in pneumococcal strains in Latin American countries

Countries	% of Strains with Indicated Gene(s)			Year(s)	Reference(s)
	*erm(B)*	*mef(A/E)*	*erm(B)+mefE*		
Argentina, Brazil, Mexico	42	56	1	1999–2000	59
Argentina	34	58	6	1993–2001	54
Colombia	56.9	40.2	7.1	1994–2008	98
Colombia	60.3	33	–	2005–2008	57
Venezuela	79.1	12.5	4.2	2007	86
Peru	30.9	47.6	19.1	2006–2008*^†^	71
Peru	48	0	48	2006–2008*	71
Brazil	36	44	20	2007–2012	67
Brazil	13	2	1	2008–2009^‡^	7, 99
Argentina	19.2	76.9	3.9	2009–2010	87
Peru	19.1	4.8	76.2	2009–2011*	71
Brazil	30	46	23	2010–2012	68
Brazil	14	5	8	2012–2013^§^	7, 99
Peru	29.8	7.5	55.2	2016–2019^‖^	71
Peru	31.7	31.7	18.3	2018–2019^†^^‖^	71

* Pre-PCV strains.

^†^ Carriage strains.

^‡^ IPD isolates in the pre-PCV10 period in Brazil.

^§^ IPD isolates in the post-PCV10 periods in Brazil.

^‖^ Post-PCV13 strains.

In Argentina, between 2009 and 2010, 76.9% of macrolide-resistant isolates carried *mef(A/E)/mel*, 19.2% carried the *ermB* gene, and 3.9% exhibited the *mef(A/E)*/*mel* plus *ermB* genotype.^87^ In the years 2007 to 2012 in Brazil, 44% of *S. pneumoniae *isolates carried *mef(A/E)/mel*, 36% carried the *ermB* gene, and 20% of the isolates carried the dual resistance mechanism with the *mef(A/E)/mel* plus *ermB* genotype.^67^

## PNEUMOCOCCAL SEROTYPES AND GENES ASSOCIATED WITH MACROLIDE RESISTANCE

There are more than 100 different capsular types described that vary in pathogenicity, geographical distribution, and antibiotic resistance mechanism, including macrolide resistance.^88^^,^^89^

Regarding the serotypes of the strains in Colombia, the following distribution of serotypes and genotypes of *S. pneumoniae *was observed between the years 1994 to 2008. The majority (86.5%) of serotype 6B strains carried *mefA* and 10% carried *ermB*. In contrast, only 12.5% of serotype 6A strains were positive for *mefA*, while 87.5% of them carried *ermB*. Another group of vaccine strains, those of serotype 14, were positive for *mefA* in 65.2% and for *ermB* in 30.4%; those of serotype 19F were positive for *mef(A)* in 50% and for *erm(B)* in 40%; those of serotype 19A were positive for *mef(A)* in 77.7% and for *erm(B)* in 33.3%; those of serotype 23F were positive for genotype *mef(A)* in 100%; and those of serotype 9V were positive for *mef(A)* in 33.3% and for *erm(B)* in 33.3%.^57^

In the case of Ecuador, which is the country that has the most current data, serotypes 6A/D/C, 19A, and 10A stand out because they had a higher percentage of isolation in a study in indigenous Kichwa communities of Otavalo.^90^

## FUTURE RESEARCH PERSPECTIVES ON MACROLIDE RESISTANCE IN LATIN AMERICA

Despite macrolides being a mainstay in pneumococcal disease treatment, global resistance rates are concerningly on the rise. However, epidemiological data on macrolide resistance and the mechanisms behind its dissemination in Latin American countries are limited. Research in this area is essential to help curb the antibiotic resistance pandemic. Establishing baseline information on resistance prevalence, understanding specific mechanisms in different geographic regions, and standardizing methods for studying macrolide resistance in Latin America are crucial. These efforts would provide a solid foundation for collaborative initiatives aimed at tackling the escalating antibiotic resistance crisis.

One of the main lessons learned from the current situation of macrolide resistance in Latin America is that the trend of macrolide resistance is on an upward trajectory. Some reports, particularly from Peru, show ∼80% resistance to macrolides in *S. pneumoniae *strains isolated from IPD cases.^71^ Research addressing the mechanistic basis of the acquisition or transfer of macrolide resistance among strains, as well as surveillance studies, is needed to develop intervention strategies and implement evidence-based public health policies for the use of macrolide antibiotics in the region.

The increase in prevalence of *S. pneumoniae *strains carrying both Mega and *ermB* is an interesting biological phenomenon. The *mef(E)*/*mel* genes and/or *ermB* are often carried in integrative and conjugative elements (ICEs) of the Tn*916*-related family and the Tn*5253*-related family, which also carry the *tetM* gene for resistance to tetracycline.^91^^–^^93^ The Tn*916*-related elements carrying Mega inserted into *orf6* are termed Tn*2009*, whereas those carrying only *ermB* are named Tn*6002* and Tn*916*-related elements carrying both Mega and *ermB* are often called Tn*2010* elements.^92^^,^^94^ Although we lack specific genetic evidence of Tn*916*-related elements in the *S. pneumoniae *strains isolated in Latin America discussed in this review, we can certainly speculate that the majority of *S. pneumoniae *strains carrying Mega also carry Tn*2009* and that those strains carrying Mega and *ermB,* whose prevalence has increased in the last few years, carry these genes in Tn*2010* elements.

So, why do *S. pneumoniae *strains need two mechanisms for macrolide resistance? The selection of these strains in the human host is obvious, because of their increased prevalence in IPD cases. We have recently demonstrated that the acquisition of pneumococcal ICEs is facilitated by the transformation machinery.^95^ Because Tn*916*-related elements are similar, one would expect that the frequency of acquisition of either Tn*2009* or Tn*2010* in nature is similar. If this were to occur, and macrolide selection is the only driver of selection, then the prevalence of strains carrying one element, or both, should be similar. Because this phenomenon does not appear to occur, we hypothesize that selection towards *S. pneumoniae *strains carrying Tn*2010*-like elements (i.e., carrying both Mega and *ermB*) is driven by an additional fitness cost conferred by the presence of both elements. This hypothesis is currently under investigation in our laboratories.

Finally, the current information available about the macrolide resistance situation in Latin America should be considered by regional public health authorities to make recommendations on what can be done to prevent the growth and spread of antibiotic resistance. For instance, considering the current trends in macrolide resistance prevalence, it is recommended that over-the-counter sale of antibiotics be avoided and that public health educational campaigns for proper use and adherence to antibiotic treatment be improved. More specifically, protocols for the clinical use of macrolides such as azithromycin for treating CAP should be revised and updated.
